# Insect hypersensitivity beyond bee and wasp venom allergy 

**DOI:** 10.5414/ALX02123E

**Published:** 2020-11-30

**Authors:** Wolfgang Hemmer, Felix Wantke

**Affiliations:** Floridsdorf Allergy Center, Vienna

**Keywords:** blood-feeding insects, mosquito allergy, mosquitoes, horse flies, black flies, biting midges, salivary allergens

## Abstract

The bites of blood-feeding insects regularly induce sensitization to salivary proteins and cause local hypersensitivity reactions in over 90% of the population, representing either an IgE-mediated immediate wheal and flare reaction or a T cell-driven delayed papule. Long-lasting large local reactions and bullous reactions may cause significant discomfort and reduction in quality-of-life. Anaphylaxis is rarely reported though proven for several insects, above all mosquitoes, horse flies, and kissing bugs. Recently, salivary gland proteins have been thoroughly studied in some blood-feeding insect species, and several allergens have been identified. Interestingly, many of them belong to the same protein families as the well-known honeybee and wasp venom allergens (phospholipases, hyaluronidases, antigens 5, serine proteases) though sequence identities are mostly low. There is still insufficient evidence for the proposed cross-reactivity between salivary proteins from blood-feeding insects and Hymenoptera venom allergens.

**German version published in Allergologie, Vol. 43, No. 2/2020, pp. 73-81**

## Introduction 

In contrast to bees and wasps, blood-feeding insects get less attention in allergological practice, because their bites primarily cause cutaneous hypersensitivity reactions and rarely anaphylaxis. In addition, the usually mild bite reactions are often believed to be infections or toxic by nature, although they actually represent true immune reactions. The borderline between “normal” and “allergic” is vague. 

## Cutaneous immediate and late reaction as ubiquitous reaction patterns 

The insect saliva secreted during the bite contains biogenic amines and vasoactive peptides as well as various anticoagulant proteins and digestive enzymes, which regularly induce an antigen-specific immune response in the host [33, 49]. Some saliva components have been shown to have an immunomodulatory effect in terms of Th2 polarization, possibly as an evolutionary strategy to complete blood meal more rapidly through inducing local vasodilation [3]. 

**Figure 1.**
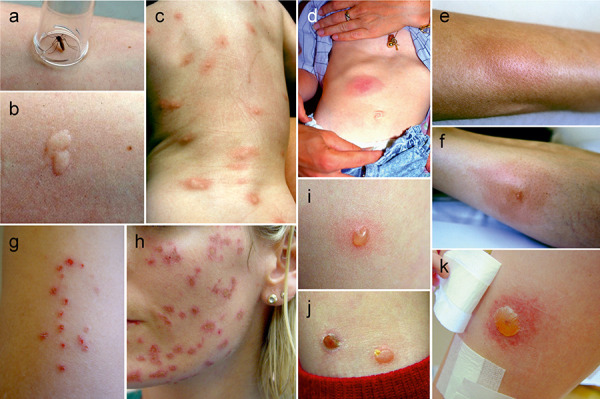
Skin reactions after bites by hematophagous insects. a, b: Immediate wheal and flare reaction during a bite provocation with a mosquito, “double” wheal after interruption of blood feeding; c: Type 4 late reactions (indurated papules) after multiple mosquito bites in an infant; d, e, f: Large local reactions after mosquito bites; g: Pearl-like arranged skin lesions caused by fleas bites; h: Bacterial superinfections due to scratching after multiple mosquito bites; i, j: Bullous skin reactions caused by flea bites after 3 (i) and 5 (j) days; k: Massive bullous bite reaction after a suspected black fly bite.

In the majority of people, either a transient immediate cutaneous reaction characterized by wheals, erythema, and itching occurs within a few minutes at the bite site (immediate reaction), or an indurated papule persisting for several days develops after 12 – 24 hours (late reaction) (Figure 1a, b, c). In Central Europe, > 70% of the population develops a cutaneous immediate reaction after mosquito bites, 50 – 60% a late reaction, and 40% both [13]. Similar values have been reported from Asia and Central America [11, 22]. 

The immediate wheal-and-flare reaction has been shown to represent a localized IgE-mediated type 1 reaction [23, 33]. In addition to histamine, probably also capsaicin-sensitive sensory neurons play an essential role in bite-associated itching [29]. The heterogeneous delayed reactions have been little studied thus far. They probably represent primarily T-cell-dependent type 4 reactions and show non-specific infiltrates with macrophages, neutrophils, eosinophils and CD4+/CD8+ T lymphocytes. The saliva allergens are already secreted by the insect during probing, so that skin reactions occur even if the feeding process is interrupted (Figure 1a, b). 

Cutaneous reactivity patterns often show a characteristic chronological sequence of different reaction stages, starting with no reaction and progressing via delayed reaction and immediate-type reaction to secondary non-responsiveness (Table 1). Upon average exposure, 80 – 90% of children and adults remain in stages 2 – 4 for decades [30]. Only ~ 5% achieve secondary tolerance [13, 22, 26], especially in case of a very high number of insect bites such as e.g. in Lapland, where up to 50% of the population become non-reactive [41]. The stage concept, which is largely based on epidemiological observations, was also experimentally proven in a volunteer receiving a total of 2,000 mosquito bites over a 10-months period [33]. 

## Large local reactions 

Severe skin reactions in terms of delayed swellings up to > 10 cm in diameter, blisters or necroses are found in 3 – 10% (Figure 1d, e, f, i, j, k) [11, 13]. The pathomechanisms underlying these reactions are poorly understood. In some cases, skin reactions may be accompanied by general symptoms like fever and lymphadenopathy (“Skeeter syndrome”) [45]. Superinfections are not uncommon to occur (Figure 1h) and may require antibiotic therapy. 

Papular urticaria is a chronic generalized cutaneous hypersensitivity reaction that is seen especially in tropical regions in children after mosquito, bug, and flea bites [10]. It is characterized by multiple, disseminated urticarial and papular lesions and is probably due to the restimulation of resident T cells following new bites. 

Differential diagnosis of allergic hypersensitivity includes severe bullous necrotic ulcerations associated with general symptoms after mosquito bites in patients with NK-cell lymphocytosis and chronic EBV infection [2, 19]. This condition was described almost exclusively in children from East Asia. EBV reactivation in infected NK cells by mosquito-specific CD4+ T cells has been discussed as the underlying pathomechanism. Similar exaggerated local reactions have been observed also in other immune diseases affecting T-cell immunity (e.g. CML, AIDS). 

## Systemic reactions 

Anaphylactic reactions are rare, possibly because of the small amount of antigen injected by blood-feeding insects. The most frequently reported triggers are horse flies and kissing bugs [14, 20, 25, 28, 38, 42], whose salivary glands contain ~ 10 – 30 times more protein than those of mosquitoes [47]. Anaphylaxis has also been credibly documented after bites from mosquitoes, tsetse flies, and louse flies [32, 40]. A possible risk factor for anaphylactic reactions may be the presence of mastocytosis [27, 40], which is also a well-documented risk factor for severe anaphylaxis in Hymenoptera allergy. 

## Relevant insect species 

**Figure 2.**
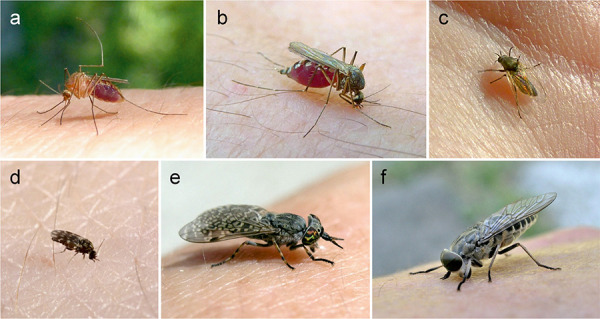
Central European blood-feeding insects. a: *Culex pipiens* (Culicidae, mosquitoes), b: *Aedes vexans* (Culicidae, mosquitoes), c: *Simulium *spp. (Simuliidae, black flies), d: *Culicoides *spp. (Ceratopogonidae, biting midges), e: *Haematopota pluvialis*, Common horse fly, cleg fly (Tabanidae, horse flies), f: *Tabanus bromius*, band-eyed brown horse fly (Tabanidae, horse flies). Photos a, b, c, e, f: W. Hemmer, d: https://www.scienceimage.csiro.au/image/11052.

Most hematophagous insects causing allergic reactions belong to the order Diptera (mosquitoes and flies) (Table 2). Among them, the mosquitoes (Culicidae) are most important. In Central Europe, ~ 50 different species are known. 10 – 20% of them may play a role as relevant pests due to high abundance, above all species from the genera *Aedes* and *Culex* (Figure 2a, b). The species composition of mosquitoes varies locally and seasonally, which is clinically important since cross-reactivity between different species may be limited [5, 34]. 

In Central Europe, stings by horse flies are most frequently caused by *Haematopota* species (Common horse fly, cleg fly) (Figure 2e), and less frequently by the much larger *Tabanus* species (giant horse flies) (Figure 2f). *Chrysops* species (deer flies) occasionally sting humans and may be confused with wasps because of marked black and yellow color patterns on their abdomen. 

The 2 – 5 mm small, diurnal black flies (Simuliidae) (Figure 2c), whose larvae develop in flowing waters, can occur in large numbers locally. The persistent delayed reactions after multiple bites cause the clinical picture known as “simuliosis”. Both, the black flies as well as the minute “biting midges” (Ceratopogonidae) (Figure 2d), which are only 0.5 – 3 mm in size, have been intensively studied as the cause of summer eczema (“sweet itch”) in horses [21]. Many Ceratopogonidae species also parasitize on humans and can cause cutaneous hypersensitivity reactions of type 1 and 4 [8]. 

Among the bugs, bedbugs (Cimicidae) and kissing bugs (Reduviidae) are of importance. Bedbugs (*Cimex lectularius*) only sting at night. They often cause bullous, slow-healing lesions with the histological findings of necrotizing eosinophilic vasculitis [24]. Kissing bugs from the subfamily Triatominae (e.g. *Triatoma protracta*) are known in Central America and the southern USA as the cause of sometimes severe anaphylactic reactions. They are 1 – 3 cm in size and primarily parasite on small mammals but occasionally also on humans. Their bites are painful only when interrupted and typically occur at night (“nocturnal anaphylaxis”) [20, 28]. 

## Are some people particularly attractive to mosquitoes? 

The claim that some people may be particularly attractive to mosquitoes can be supported by numerous scientific studies [7, 39]. For far distance localization of potential hosts, blood-feeding insects use visual stimuli and especially carbon dioxide gradients. Differences in concentrations as low as 0.015% can be perceived by the insects. For close-up orientation (< 1 m), differences in temperature and humidity as well as the specific “skin odor”, which is a complex mixture of ammonia, lactic acid, acetic acid, and various fatty acids, come into play. This skin odor varies individually and is determined by genetic factors as well as by the cutaneous microflora. An increased attractiveness for mosquitoes has been described for persons with blood group AB or atopic dermatitis, among others. Pregnancy, heavy sweating, and beer consumption are also said to increase the attractiveness (for mosquitoes). Age and sex have little influence. 

## Allergens in insect saliva: the “sialome” of blood-feeding insects 

Many identified allergens in insect saliva represent ubiquitous proteins or structurally conserved digestive enzymes found in many species, e.g., antigen 5-like proteins, hyaluronidases, apyrases, maltases, and amylases [6]. The diversity of proteins that are specifically involved in blood-feeding is greater because hematophagy evolved several times independently within the arthropods, and group-specific proteins have evolved in adaptation to different hosts [6]. For example, three major allergens are known from the yellow fever mosquito *Aedes aegypti* (Aed a 1 – 3): Aed a 1 is an apyrase enzyme as commonly found in many insects, Aed a 2 is a D7 protein only found within the insect order Diptera, and Aed a 3 is an “aegyptin” protein restricted to mosquitoes within the Diptera. Among mosquitoes, even further evolutionary differentiations can be observed: after the extinction of the dinosaurs, the genera *Aedes* and *Anopheles* specialized in parasitizing on mammals and subsequently developed specific saliva components optimized for the mammalian coagulation system which are not found in the genus *Culex* having specialized in feeding on birds [9]. This knowledge is important for understanding why cross-reactions may occur between some saliva proteins of distantly related insect groups while other allergens are family- or even genus-specific. 

Relevant saliva allergens were first identified in mosquitoes after isolated salivary glands or pure mosquito saliva have been used as allergen source instead of whole body extracts [5, 34]. Allergens have been extensively studied in the yellow fever mosquito *Aedes aegypti*. Four allergens have been cloned and recombinantly produced, but are not commercially available for routine diagnostics [35, 36]. Three allergens (hyaluronidase, antigen 5, apyrase) were recently identified in the Asian horse fly species *Tabanus yao* [25]. They show up to 40% sequence identity with homologous proteins from mosquitoes and Hymenoptera venoms. 

Studies on allergens from black flies and biting midges come primarily from the veterinary field since these insects commonly cause severe dermatitis in horses [44, 46]. Interestingly, the saliva allergens recognized by allergic horses are not identical to those recognized by allergic humans [8]. Accordingly, data from veterinary studies may not always be transferable to humans. The main saliva allergens identified in the kissing bug *Triatoma protracta* and in the pigeon tick *Argas reflexus* are members of the lipocalin family [31]. Their sequence similarity with the lipocalins involved in respiratory allergy to furry animals (e.g. Can f 1, Fel d 4) is only low however. 

## How important is allergy diagnosis? 

The diagnosis of insect bite hypersensitivity is primarily based on medical history. A reliable identification of the culprit insect having caused a skin reaction is rarely possible. Pearl-like serial lesions (“breakfast, lunch, and dinner”) and bullous reactions indicate bites by fleas or bed bugs [37] (Figure 1g, i, j), which may suggest appropriate eradication measures. 

In view of the high sensitization rates in the general population and the manifold pathomechanisms underlying skin reactions, the importance and feasibility of allergy diagnostics may be limited. It is currently unknown if diagnostic tests would be able to reliably discriminate between “normal” reactors and those with exaggerated skin hypersensitivity reactions or risk of anaphylaxis. Moreover, it is not known how many and which particular insect species have to be considered for diagnostics since the range of locally relevant insects varies significantly. 

Currently, a very limited number of assays using whole body extracts from mosquitoes and horse flies exist for in vitro diagnosis. Skin test extracts are hardly available. The sensitivity of these extracts is low because relevant salivary gland proteins are under-represented in them. Also their specificity is low because IgE binding is often due to non-salivary proteins (e.g., tropomyosin) or due to CCDs. Therefore, any positive blood test result has to be interpreted with caution. Recombinant allergens currently are not commercially available. 

## Therapy 

Topical antihistamines are widely used for cutaneous immediate reactions, yet they have no proven effect. In contrast, premedication with oral antihistamines (levocetirizine, cetirizine, loratadine, rupatadine) in standard daily doses can significantly reduce itching and wheal size in children and adults [17, 18]. Treating delayed cutaneous reactions with topical corticosteroids appears reasonable, but controlled studies do not exist. Immunotherapy trials have been carried out sporadically in the past and recently with mixed success [1, 4]. 

An important measure in insect bite hypersensitivity is consistent bite prophylaxis by using insect nets and repellents. The still most effective repellents are diethyltoluamide (DEET) and icaridin/picaridin. The latter is also suitable for infants aged 2 years and older, whereas DEET may sometimes cause side effects [48]. Maximal protection is achieved by combining repellent use plus insecticidal impregnation of clothes and/or insect nets with permethrin. There is no good proof of efficacy for many alternative methods that are commonly propagated (vitamin B, ultrasound devices, etc.) [12]. 

## Possible relationships to bee and wasp venom allergy 

Based on clinical observations and co-sensitization patterns, cross-reactivity between horse flies/mosquitoes and Hymenoptera has been repeatedly suspected. Such cross-reactivity has been proposed to occur with wasps (“wasp-mosquito-horsefly-syndrome”) [38, 42] and more recently also with honeybee (“bee-mosquito-syndrome”) [43]. In fact, proteomic data confirm that homologues of many important Hymenoptera venom allergens do occur also in the saliva of hematophagous insects, but there is still insufficient experimental evidence substantiating the postulated cross-reactivity and its potential clinical relevance. Antigen 5-like proteins have been identified as allergens in the saliva of horse flies, biting midges, black flies, fleas, and the tsetse fly (Table 3). However, the sequence identities with Ves v 5 from wasp venom are always less than 30% which unlikely will lead to relevant cross-reactivity. Phospholipases showing 35 – 45% sequence identity with wasp Ves v 1 and honeybee Api m 1 are known from several mosquitoes and sand flies, but they have not yet been recognized as allergens in these species, and their presence in saliva is uncertain. The same is true for serine proteases known from various mosquitoes, midges, and kissing bugs and showing 30 – 45% homology to Api m 7 from honeybee and Pol d 4 from the paper wasp *Polistes dominula* [15]. 

The most likely allergen mediating cross-reactivity between blood-feeding insects and Hymenoptera is hyaluronidase [38, 42]. Hyaluronidases are proteins that have been strongly conserved in sequence and structure during evolution. They have been recognized as relevant saliva allergens in horse flies and biting midges and are readily detectable in the salivary glands of most other blood-feeding insects, too [15, 47] (Table 3). The sequence identity with the hyaluronidases from wasp (Ves v 2) and honeybee (Api m 2) may be as high as 60%, which is comparable to the similarity between Ves v 2 and Api m 2 themselves. Since hyaluronidases are highly glycosylated, studies investigating cross-reactivity have to consider cross-reactivity due to CCDs. In a recent study from China, recombinant hyaluronidase from the horse fly *Tabanus yao* (rTab y 2) could not significantly inhibit IgE binding to crude wasp venom even at very high allergen concentrations [25]. However, reciprocal inhibition was not performed and therefore no final conclusions can be drawn from these data. Even in case of significant cross-reactivity between hyaluronidases from insect saliva and venoms it should be kept in mind that hyaluronidases represent only minor allergens in Hymenoptera venom allergy [16]. 

## Concluding remarks 

Though the majority of people shows some kind of cutaneous hypersensitivity after bites from blood-feeding insects, these reactions are usually mild and of limited importance in everyday allergy practice. The pathomechanisms underlying large local and bullous reactions are still poorly studied. Concomitant mastocytosis may be a risk factor for anaphylactic reactions which are rare but have been credibly documented for various hematophagous insects. The currently available diagnostic tools are poor. Therapy focuses on avoidance measures along with antihistamine premedication and symptomatic treatment. In the recent past, numerous allergens from the salivary glands of various insect groups have been identified, which partly belong to the same protein families as the well-known allergens from bee and wasp venom. The proposed cross-reactivity between saliva proteins and Hymenoptera venom allergens is still obscure and needs to be clarified in future molecular studies. 

## Funding 

No funding. 

## Conflict of interest 

The authors declare that there is no conflict of interest. 

**Table 1.** Stage theory of the progression of cutaneous hypersensitivity reactions to bites from hematophagous insects.

Stage	Immediate reaction	Delayed reaction
1 (naive)	–	–
2	–	+
3	+	+
4	+	–
5 (tolerance)	–	–

**Table 2.** Blood-feeding insects causing allergic reactions.

Scientific name	English name	Important genera	c, s
Diptera (flies and midges)			
Nematocera (midges)			
Culicidae	mosquitoes	*Aedes, Culex, Anopheles*	c, s
Simuliidae	black flies	*Simulium*	c, s?
Ceratopogonidae	biting midges	*Culicoides, Forcipomyia*	c
Phlebotomidae	sand flies	*Phlebotomus, Lutzomyia*	c
Brachycera (flies)			
Tabanidae	horse flies, deer flies	*Haematopota*, *Tabanus, Chrysops*	c, s
Glossinidae	tsetse flies	*Glossina*	c, s
Muscidae	house/stable flies	*Stomoxys*	c
Hippoboscidae	louse flies, keds	*Hippobosca, Lipoptena*	c, s
Siphonaptera (fleas)			
Pulicidae	fleas	*Pulex, Ctenocephalides*	c
Heteroptera (bugs)			
Reduviidae	kissing bugs	*Triatoma*	c, s
Cimicidae	bed bugs	*Cimex lectularius*	c

c: cutaneous reactions; s: systemic reactions.

**Table 3.** Occurrence of proteins/allergens with homology to known Hymenoptera venom allergens in blood-feeding insects.

	Antigen 5-like	Hyaluroni-dase	PLA_1_	PLA_2_	Serine protease
Honeybee	–	Api m 2	–	Api m 1	Api m 7
Wasps	Ves v 5	Ves v 2	Ves v 1	–	Pol d 4
Mosquitoes	+	+	+	+	+
Black flies	++	+			
Biting midges	++	++			++
Sand flies		+		+	
Horse flies	++	++			
Tsetse flies	++				
Fleas	++	+			
Kissing bugs	+				+

+ = detected as protein; ++ = identified as allergen; PLA = phospholipase A.
